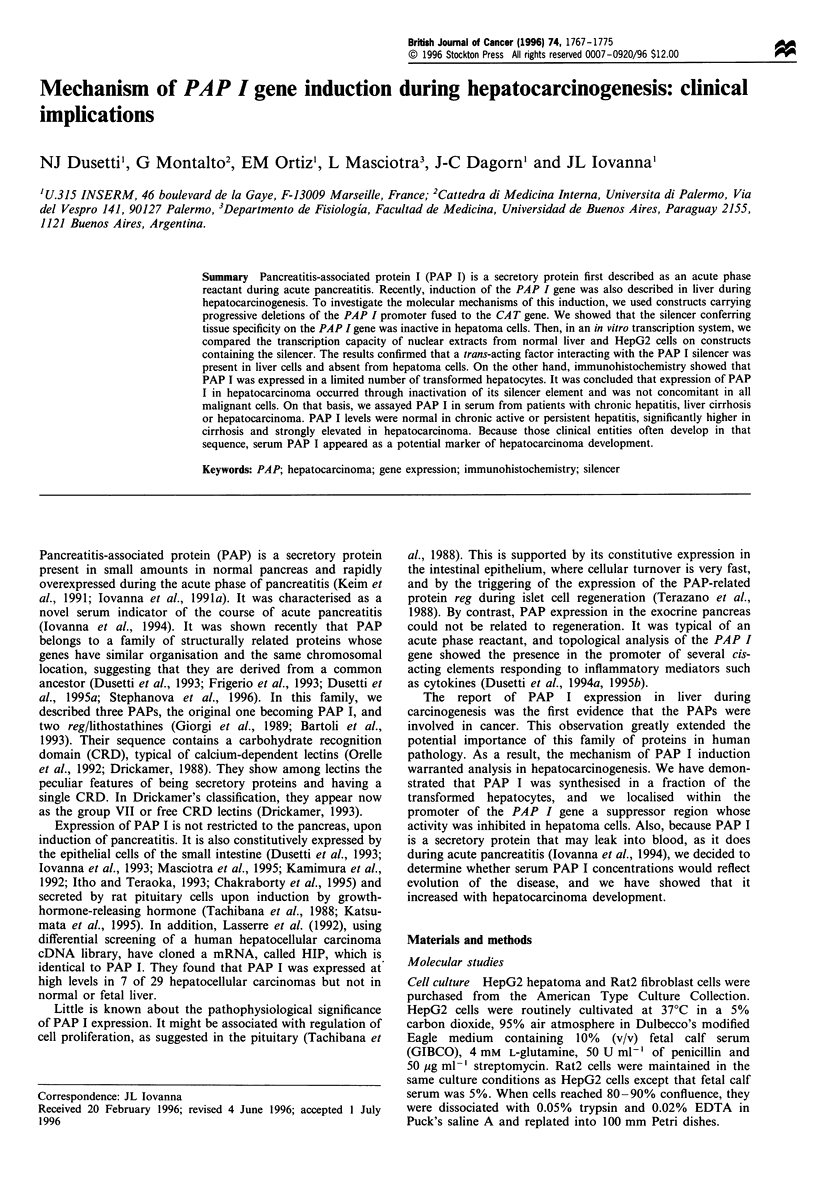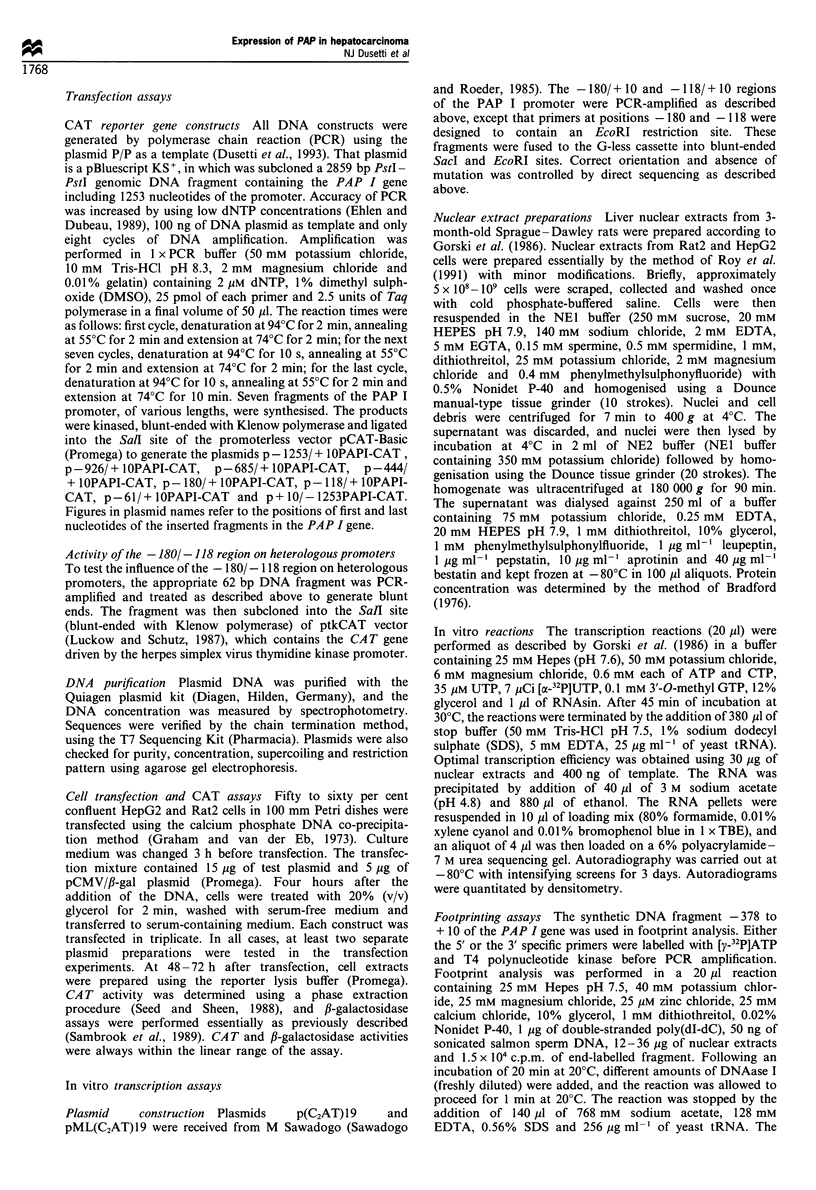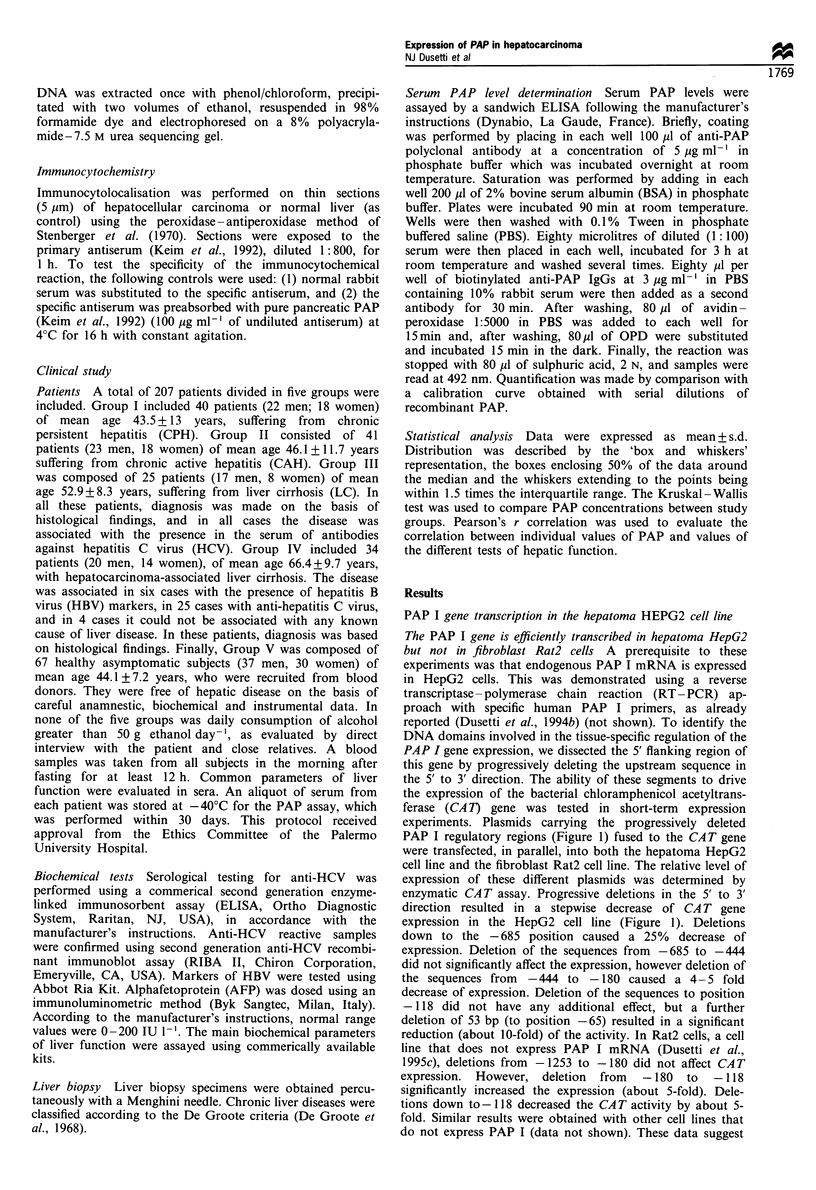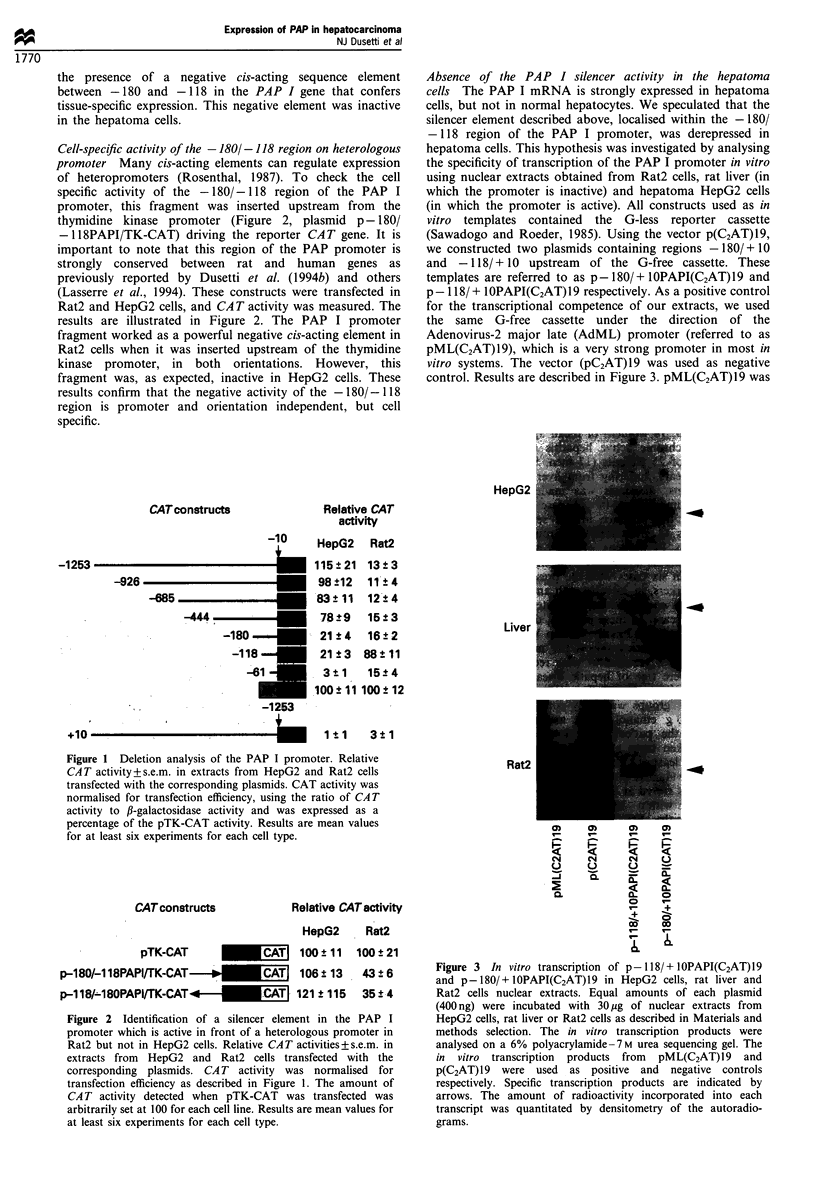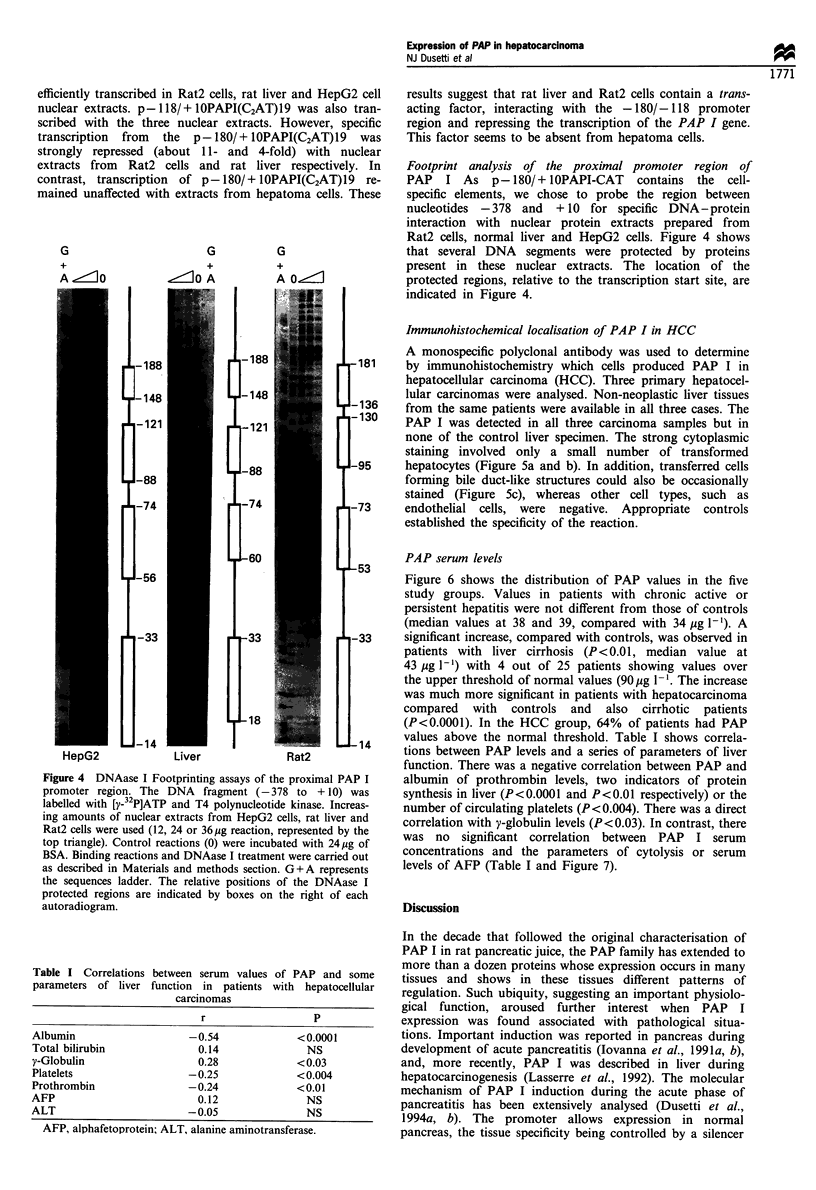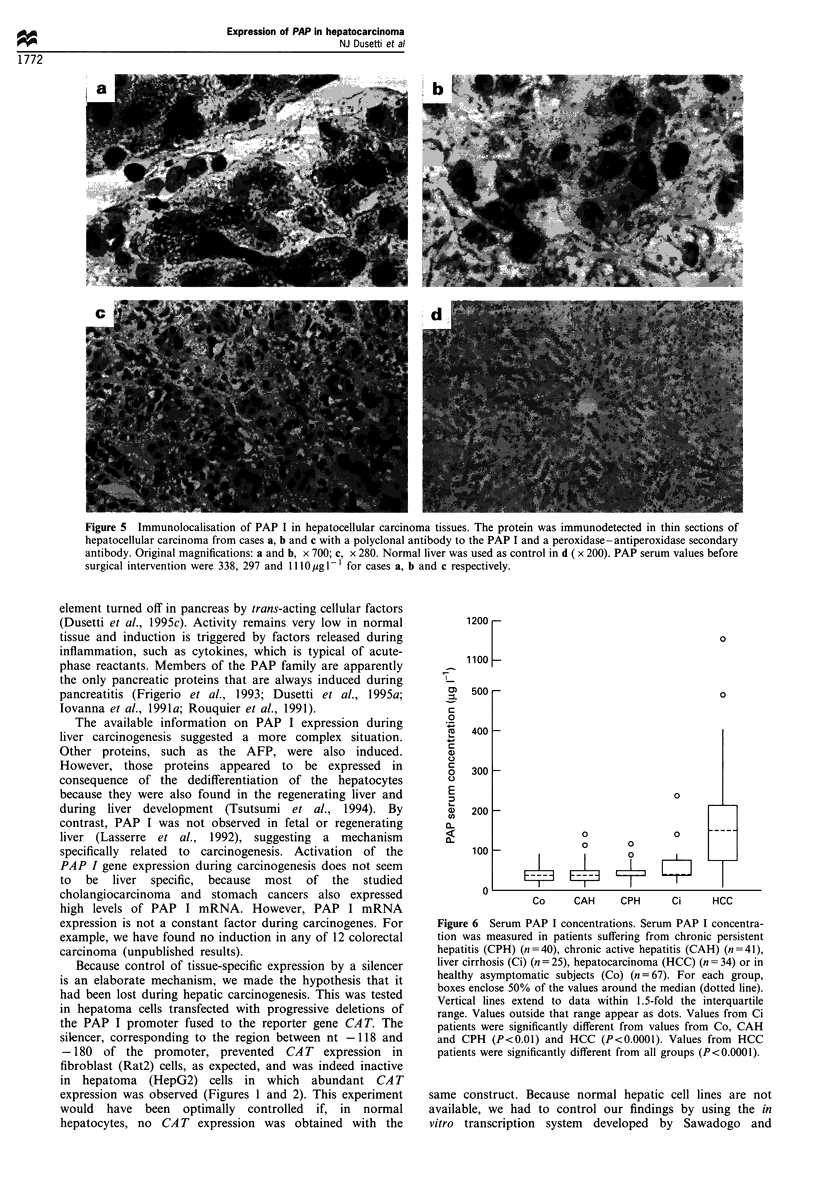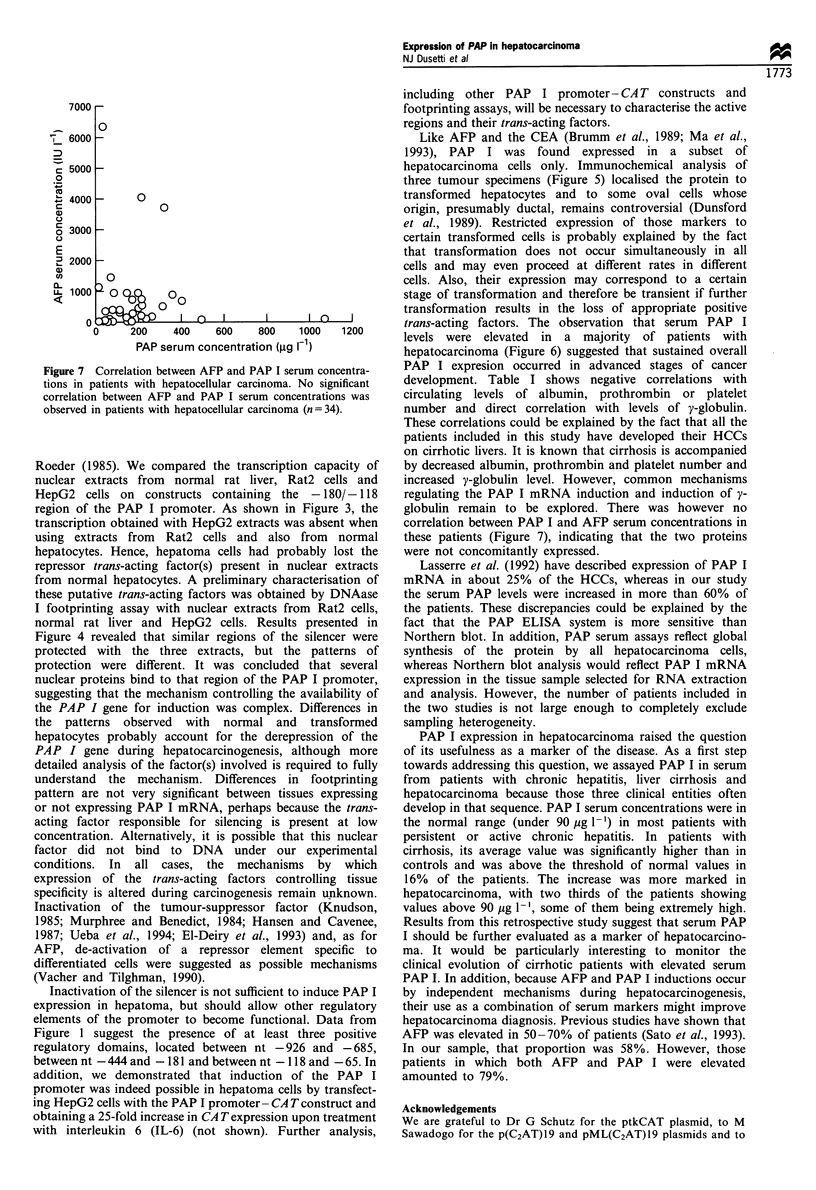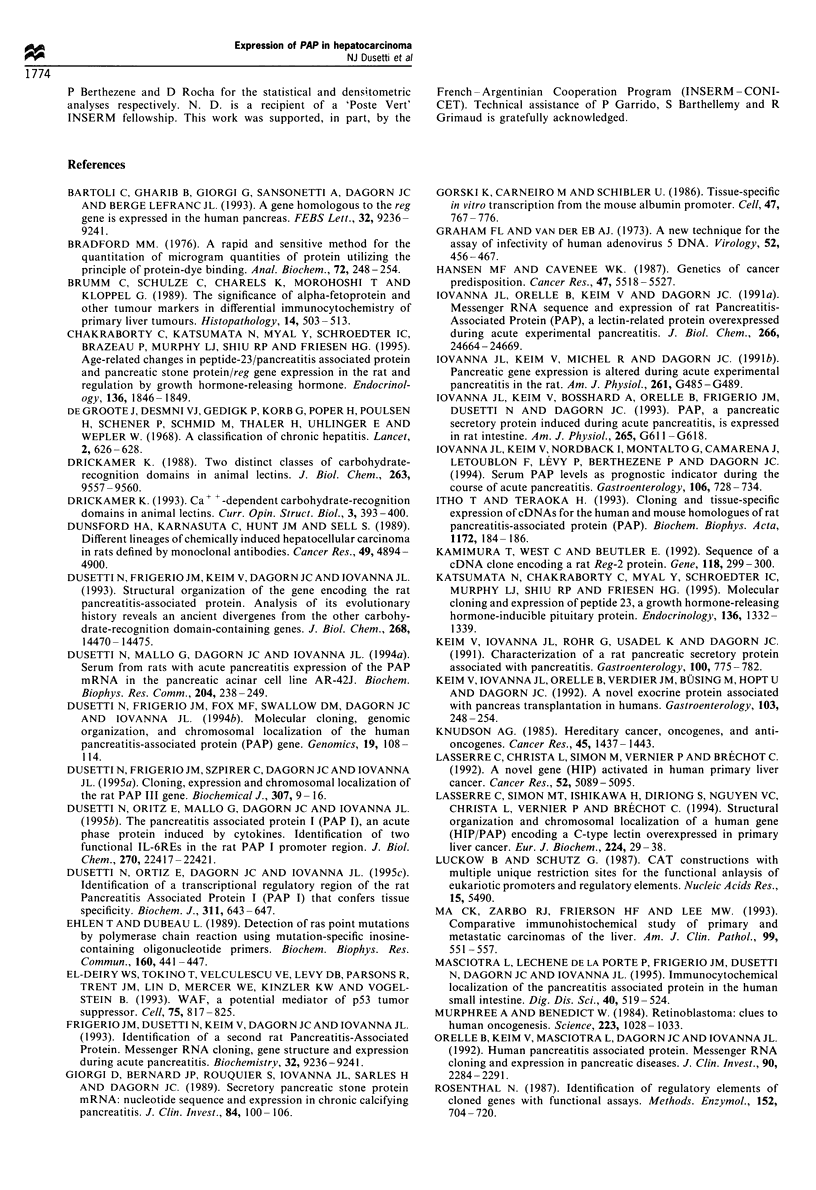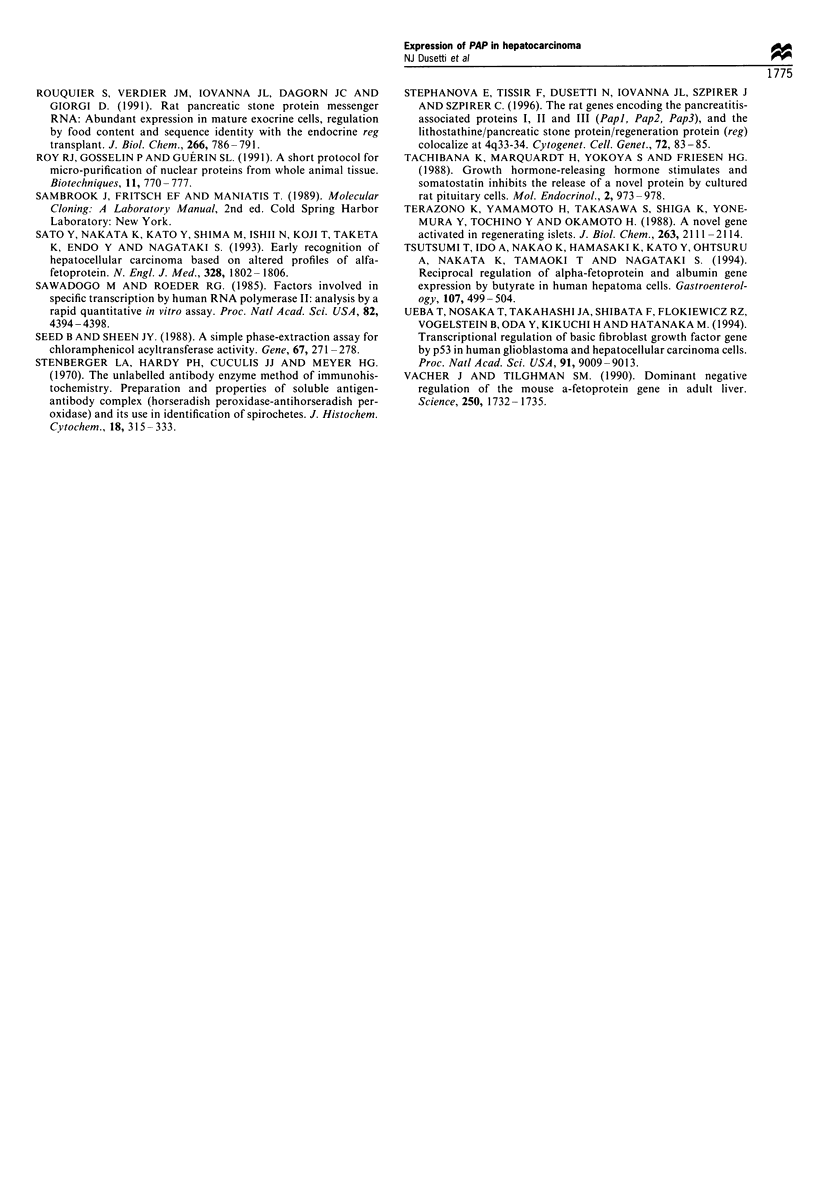# Mechanism of PAP I gene induction during hepatocarcinogenesis: clinical implications.

**DOI:** 10.1038/bjc.1996.628

**Published:** 1996-12

**Authors:** N. J. Dusetti, G. Montalto, E. M. Ortiz, L. Masciotra, J. C. Dagorn, J. L. Iovanna

**Affiliations:** U.315 INSERM, Marseille, France.

## Abstract

**Images:**


British Journal of Cancer (1996) 74, 1767-1775
© 1996 Stockton Press All rights reserved 0007-0920/96 $12.00

### Mechanism of *PAP I* gene induction during hepatocarcinogenesis: clinical implications

NJ Dusetti[1], G Montalto[2], EM Ortiz[1], L Masciotra[3], J-C Dagorn[1] and JL Iovanna[1]

*[1]U.315 INSERM, 46 boulevard de la Gaye, F-13009 Marseille, France; [2]Cattedra di Medicina Interna, Universita di Palermo, Via del Vespro 141, 90127 Palermo, [3]Departmento de Fisiologia, Facultad de Medicina, Universidad de Buenos Aires, Paraguay 2155, 1121 Buenos Aires, Argentina.*

**Summary** Pancreatitis-associated protein I (PAP I) is a secretory protein first described as an acute phase reactant during acute pancreatitis. Recently, induction of the *PAP I* gene was also described in liver during hepatocarcinogenesis. To investigate the molecular mechanisms of this induction, we used constructs carrying progressive deletions of the *PAP I* promoter fused to the *CAT* gene. We showed that the silencer conferring tissue specificity on the *PAP I* gene was inactive in hepatoma cells. Then, in an *in vitro* transcription system, we compared the transcription capacity of nuclear extracts from normal liver and HepG2 cells on constructs containing the silencer. The results confirmed that a *trans*-acting factor interacting with the PAP I silencer was present in liver cells and absent from hepatoma cells. On the other hand, immunohistochemistry showed that PAP I was expressed in a limited number of transformed hepatocytes. It was concluded that expression of PAP I in hepatocarcinoma occurred through inactivation of its silencer element and was not concomitant in all malignant cells. On that basis, we assayed PAP I in serum from patients with chronic hepatitis, liver cirrhosis or hepatocarcinoma. PAP I levels were normal in chronic active or persistent hepatitis, significantly higher in cirrhosis and strongly elevated in hepatocarcinoma. Because those clinical entities often develop in that sequence, serum PAP I appeared as a potential marker of hepatocarcinoma development.

**Keywords:** *PAP*; hepatocarcinoma; gene expression; immunohistochemistry; silencer

Pancreatitis-associated protein (PAP) is a secretory protein present in small amounts in normal pancreas and rapidly overexpressed during the acute phase of pancreatitis (Keim *et al.*, 1991; Iovanna *et al.*, 1991*a*). It was characterised as a novel serum indicator of the course of acute pancreatitis (Iovanna *et al.*, 1994). It was shown recently that PAP belongs to a family of structurally related proteins whose genes have similar organisation and the same chromosomal location, suggesting that they are derived from a common ancestor (Dusetti *et al.*, 1993; Frigerio *et al.*, 1993; Dusetti *et al.*, 1995*a*; Stephanova *et al.*, 1996). In this family, we described three PAPs, the original one becoming PAP I, and two *reg*/lithostathines (Giorgi *et al.*, 1989; Bartoli *et al.*, 1993). Their sequence contains a carbohydrate recognition domain (CRD), typical of calcium-dependent lectins (Orelle *et al.*, 1992; Drickamer, 1988). They show among lectins the peculiar features of being secretory proteins and having a single CRD. In Drickamer's classification, they appear now as the group VII or free CRD lectins (Drickamer, 1993).

Expression of PAP I is not restricted to the pancreas, upon induction of pancreatitis. It is also constitutively expressed by the epithelial cells of the small intestine (Dusetti *et al.*, 1993; Iovanna *et al.*, 1993; Masciotra *et al.*, 1995; Kamimura *et al.*, 1992; Itho and Teraoka, 1993; Chakraborty *et al.*, 1995) and secreted by rat pituitary cells upon induction by growth-hormone-releasing hormone (Tachibana *et al.*, 1988; Katsumata *et al.*, 1995). In addition, Lasserre *et al.* (1992), using differential screening of a human hepatocellular carcinoma cDNA library, have cloned a mRNA, called HIP, which is identical to PAP I. They found that PAP I was expressed at high levels in 7 of 29 hepatocellular carcinomas but not in normal or fetal liver.

Little is known about the pathophysiological significance of PAP I expression. It might be associated with regulation of cell proliferation, as suggested in the pituitary (Tachibana *et al.*, 1988). This is supported by its constitutive expression in the intestinal epithelium, where cellular turnover is very fast, and by the triggering of the expression of the PAP-related protein *reg* during islet cell regeneration (Terazano *et al.*, 1988). By contrast, PAP expression in the exocrine pancreas could not be related to regeneration. It was typical of an acute phase reactant, and topological analysis of the *PAP I* gene showed the presence in the promoter of several *cis*-acting elements responding to inflammatory mediators such as cytokines (Dusetti *et al.*, 1994*a*, 1995*b*).

The report of PAP I expression in liver during carcinogenesis was the first evidence that the PAPs were involved in cancer. This observation greatly extended the potential importance of this family of proteins in human pathology. As a result, the mechanism of PAP I induction warranted analysis in hepatocarcinogenesis. We have demonstrated that PAP I was synthesised in a fraction of the transformed hepatocytes, and we localised within the promoter of the *PAP I* gene a suppressor region whose activity was inhibited in hepatoma cells. Also, because PAP I is a secretory protein that may leak into blood, as it does during acute pancreatitis (Iovanna *et al.*, 1994), we decided to determine whether serum PAP I concentrations would reflect evolution of the disease, and we have showed that it increased with hepatocarcinoma development.

### Materials and methods

### *Molecular studies*

*Cell culture* HepG2 hepatoma and Rat2 fibroblast cells were purchased from the American Type Culture Collection. HepG2 cells were routinely cultivated at 37°C in a 5% carbon dioxide, 95% air atmosphere in Dulbecco's modified Eagle medium containing 10% (v/v) fetal calf serum (GIBCO), 4 mM L-glutamine, 50 U $ml^{-1}$ of penicillin and 50 $\mu g$ $ml^{-1}$ streptomycin. Rat2 cells were maintained in the same culture conditions as HepG2 cells except that fetal calf serum was 5%. When cells reached 80-90% confluence, they were dissociated with 0.05% trypsin and 0.02% EDTA in Puck's saline A and replated into 100 mm Petri dishes.

Correspondence: JL Iovanna
Received 20 February 1996; revised 4 June 1996; accepted 1 July 1996

*Transfection assays*

CAT *reporter gene constructs* All DNA constructs were generated by polymerase chain reaction (PCR) using the plasmid P/P as a template (Dusetti *et al*., 1993). That plasmid is a pBluescript $KS^+$, in which was subcloned a 2859 bp *Pst*I–*Pst*I genomic DNA fragment containing the *PAP I* gene including 1253 nucleotides of the promoter. Accuracy of PCR was increased by using low dNTP concentrations (Ehlen and Dubeau, 1989), 100 ng of DNA plasmid as template and only eight cycles of DNA amplification. Amplification was performed in 1 × PCR buffer (50 mM potassium chloride, 10 mM Tris-HCl pH 8.3, 2 mM magnesium chloride and 0.01% gelatin) containing 2 μM dNTP, 1% dimethyl sulphoxide (DMSO), 25 pmol of each primer and 2.5 units of *Taq* polymerase in a final volume of 50 μl. The reaction times were as follows: first cycle, denaturation at 94°C for 2 min, annealing at 55°C for 2 min and extension at 74°C for 2 min; for the next seven cycles, denaturation at 94°C for 10 s, annealing at 55°C for 2 min and extension at 74°C for 2 min; for the last cycle, denaturation at 94°C for 10 s, annealing at 55°C for 2 min and extension at 74°C for 10 min. Seven fragments of the PAP I promoter, of various lengths, were synthesised. The products were kinased, blunt-ended with Klenow polymerase and ligated into the *Sal*I site of the promoterless vector pCAT-Basic (Promega) to generate the plasmids p−1253/+10PAPI-CAT, p−926/+10PAPI-CAT, p−685/+10PAPI-CAT, p−444/+10PAPI-CAT, p−180/+10PAPI-CAT, p−118/+10PAPI-CAT, p−61/+10PAPI-CAT and p+10/−1253PAPI-CAT. Figures in plasmid names refer to the positions of first and last nucleotides of the inserted fragments in the *PAP I* gene.

*Activity of the −180/−118 region on heterologous promoters* To test the influence of the −180/−118 region on heterologous promoters, the appropriate 62 bp DNA fragment was PCR-amplified and treated as described above to generate blunt ends. The fragment was then subcloned into the *Sal*I site (blunt-ended with Klenow polymerase) of ptkCAT vector (Luckow and Schutz, 1987), which contains the *CAT* gene driven by the herpes simplex virus thymidine kinase promoter.

*DNA purification* Plasmid DNA was purified with the Quiagen plasmid kit (Diagen, Hilden, Germany), and the DNA concentration was measured by spectrophotometry. Sequences were verified by the chain termination method, using the T7 Sequencing Kit (Pharmacia). Plasmids were also checked for purity, concentration, supercoiling and restriction pattern using agarose gel electrophoresis.

*Cell transfection and* CAT *assays* Fifty to sixty per cent confluent HepG2 and Rat2 cells in 100 mm Petri dishes were transfected using the calcium phosphate DNA co-precipitation method (Graham and van der Eb, 1973). Culture medium was changed 3 h before transfection. The transfection mixture contained 15 μg of test plasmid and 5 μg of pCMV/β-gal plasmid (Promega). Four hours after the addition of the DNA, cells were treated with 20% (v/v) glycerol for 2 min, washed with serum-free medium and transferred to serum-containing medium. Each construct was transfected in triplicate. In all cases, at least two separate plasmid preparations were tested in the transfection experiments. At 48–72 h after transfection, cell extracts were prepared using the reporter lysis buffer (Promega). *CAT* activity was determined using a phase extraction procedure (Seed and Sheen, 1988), and β-galactosidase assays were performed essentially as previously described (Sambrook *et al*., 1989). *CAT* and β-galactosidase activities were always within the linear range of the assay.

In vitro *transcription assays*

*Plasmid construction* Plasmids $p(C_2AT)19$ and $pML(C_2AT)19$ were received from M Sawadogo (Sawadogo and Roeder, 1985). The −180/+10 and −118/+10 regions of the PAP I promoter were PCR-amplified as described above, except that primers at positions −180 and −118 were designed to contain an *Eco*RI restriction site. These fragments were fused to the G-less cassette into blunt-ended *Sac*I and *Eco*RI sites. Correct orientation and absence of mutation was controlled by direct sequencing as described above.

*Nuclear extract preparations* Liver nuclear extracts from 3-month-old Sprague–Dawley rats were prepared according to Gorski *et al*. (1986). Nuclear extracts from Rat2 and HepG2 cells were prepared essentially by the method of Roy *et al*. (1991) with minor modifications. Briefly, approximately $5 \times 10^8 – 10^9$ cells were scraped, collected and washed once with cold phosphate-buffered saline. Cells were then resuspended in the NE1 buffer (250 mM sucrose, 20 mM HEPES pH 7.9, 140 mM sodium chloride, 2 mM EDTA, 5 mM EGTA, 0.15 mM spermine, 0.5 mM spermidine, 1 mM, dithiothreitol, 25 mM potassium chloride, 2 mM magnesium chloride and 0.4 mM phenylmethylsulphonyfluoride) with 0.5% Nonidet P-40 and homogenised using a Dounce manual-type tissue grinder (10 strokes). Nuclei and cell debris were centrifuged for 7 min to 400 ***g*** at 4°C. The supernatant was discarded, and nuclei were then lysed by incubation at 4°C in 2 ml of NE2 buffer (NE1 buffer containing 350 mM potassium chloride) followed by homogenisation using the Dounce tissue grinder (20 strokes). The homogenate was ultracentrifuged at 180 000 ***g*** for 90 min. The supernatant was dialysed against 250 ml of a buffer containing 75 mM potassium chloride, 0.25 mM EDTA, 20 mM HEPES pH 7.9, 1 mM dithiothreitol, 10% glycerol, 1 mM phenylmethylsulphonylfluoride, 1 μg $ml^{-1}$ leupeptin, 1 μg $ml^{-1}$ pepstatin, 10 μg $ml^{-1}$ aprotinin and 40 μg $ml^{-1}$ bestatin and kept frozen at −80°C in 100 μl aliquots. Protein concentration was determined by the method of Bradford (1976).

In vitro *reactions* The transcription reactions (20 μl) were performed as described by Gorski *et al*. (1986) in a buffer containing 25 mM Hepes (pH 7.6), 50 mM potassium chloride, 6 mM magnesium chloride, 0.6 mM each of ATP and CTP, 35 μM UTP, 7 μCi [α-$^{32}$P]UTP, 0.1 mM 3′-*O*-methyl GTP, 12% glycerol and 1 μl of RNAsin. After 45 min of incubation at 30°C, the reactions were terminated by the addition of 380 μl of stop buffer (50 mM Tris-HCl pH 7.5, 1% sodium dodecyl sulphate (SDS), 5 mM EDTA, 25 μg $ml^{-1}$ of yeast tRNA). Optimal transcription efficiency was obtained using 30 μg of nuclear extracts and 400 ng of template. The RNA was precipitated by addition of 40 μl of 3 M sodium acetate (pH 4.8) and 880 μl of ethanol. The RNA pellets were resuspended in 10 μl of loading mix (80% formamide, 0.01% xylene cyanol and 0.01% bromophenol blue in 1 × TBE), and an aliquot of 4 μl was then loaded on a 6% polyacrylamide–7 M urea sequencing gel. Autoradiography was carried out at −80°C with intensifying screens for 3 days. Autoradiograms were quantitated by densitometry.

*Footprinting assays* The synthetic DNA fragment −378 to +10 of the *PAP I* gene was used in footprint analysis. Either the 5′ or the 3′ specific primers were labelled with [γ-$^{32}$P]ATP and T4 polynucleotide kinase before PCR amplification. Footprint analysis was performed in a 20 μl reaction containing 25 mM Hepes pH 7.5, 40 mM potassium chloride, 25 mM magnesium chloride, 25 μM zinc chloride, 25 mM calcium chloride, 10% glycerol, 1 mM dithiothreitol, 0.02% Nonidet P-40, 1 μg of double-stranded poly(dI-dC), 50 ng of sonicated salmon sperm DNA, 12–36 μg of nuclear extracts and $1.5 \times 10^4$ c.p.m. of end-labelled fragment. Following an incubation of 20 min at 20°C, different amounts of DNAase I (freshly diluted) were added, and the reaction was allowed to proceed for 1 min at 20°C. The reaction was stopped by the addition of 140 μl of 768 mM sodium acetate, 128 mM EDTA, 0.56% SDS and 256 μg $ml^{-1}$ of yeast tRNA. The

DNA was extracted once with phenol/chloroform, precipitated with two volumes of ethanol, resuspended in 98% formamide dye and electrophoresed on a 8% polyacrylamide–7.5 M urea sequencing gel.

*Immunocytochemistry*

Immunocytolocalisation was performed on thin sections (5 $\mu$m) of hepatocellular carcinoma or normal liver (as control) using the peroxidase–antiperoxidase method of Stenberger *et al.* (1970). Sections were exposed to the primary antiserum (Keim *et al.*, 1992), diluted 1:800, for 1 h. To test the specificity of the immunocytochemical reaction, the following controls were used: (1) normal rabbit serum was substituted to the specific antiserum, and (2) the specific antiserum was preabsorbed with pure pancreatic PAP (Keim *et al.*, 1992) (100 $\mu$g ml$^{-1}$ of undiluted antiserum) at 4°C for 16 h with constant agitation.

*Clinical study*

*Patients* A total of 207 patients divided in five groups were included. Group I included 40 patients (22 men; 18 women) of mean age 43.5±13 years, suffering from chronic persistent hepatitis (CPH). Group II consisted of 41 patients (23 men, 18 women) of mean age 46.1±11.7 years suffering from chronic active hepatitis (CAH). Group III was composed of 25 patients (17 men, 8 women) of mean age 52.9±8.3 years, suffering from liver cirrhosis (LC). In all these patients, diagnosis was made on the basis of histological findings, and in all cases the disease was associated with the presence in the serum of antibodies against hepatitis C virus (HCV). Group IV included 34 patients (20 men, 14 women), of mean age 66.4±9.7 years, with hepatocarcinoma-associated liver cirrhosis. The disease was associated in six cases with the presence of hepatitis B virus (HBV) markers, in 25 cases with anti-hepatitis C virus, and in 4 cases it could not be associated with any known cause of liver disease. In these patients, diagnosis was based on histological findings. Finally, Group V was composed of 67 healthy asymptomatic subjects (37 men, 30 women) of mean age 44.1±7.2 years, who were recruited from blood donors. They were free of hepatic disease on the basis of careful anamnestic, biochemical and instrumental data. In none of the five groups was daily consumption of alcohol greater than 50 g ethanol day$^{-1}$, as evaluated by direct interview with the patient and close relatives. A blood samples was taken from all subjects in the morning after fasting for at least 12 h. Common parameters of liver function were evaluated in sera. An aliquot of serum from each patient was stored at −40°C for the PAP assay, which was performed within 30 days. This protocol received approval from the Ethics Committee of the Palermo University Hospital.

*Biochemical tests* Serological testing for anti-HCV was performed using a commerical second generation enzyme-linked immunosorbent assay (ELISA, Ortho Diagnostic System, Raritan, NJ, USA), in accordance with the manufacturer's instructions. Anti-HCV reactive samples were confirmed using second generation anti-HCV recombinant immunoblot assay (RIBA II, Chiron Corporation, Emeryville, CA, USA). Markers of HBV were tested using Abbot Ria Kit. Alphafetoprotein (AFP) was dosed using an immunoluminometric method (Byk Sangtec, Milan, Italy). According to the manufacturer's instructions, normal range values were 0–200 IU l$^{-1}$. The main biochemical parameters of liver function were assayed using commerically available kits.

*Liver biopsy* Liver biopsy specimens were obtained percutaneously with a Menghini needle. Chronic liver diseases were classified according to the De Groote criteria (De Groote *et al.*, 1968).

*Serum PAP level determination* Serum PAP levels were assayed by a sandwich ELISA following the manufacturer's instructions (Dynabio, La Gaude, France). Briefly, coating was performed by placing in each well 100 $\mu$l of anti-PAP polyclonal antibody at a concentration of 5 $\mu$g ml$^{-1}$ in phosphate buffer which was incubated overnight at room temperature. Saturation was performed by adding in each well 200 $\mu$l of 2% bovine serum albumin (BSA) in phosphate buffer. Plates were incubated 90 min at room temperature. Wells were then washed with 0.1% Tween in phosphate buffered saline (PBS). Eighty microlitres of diluted (1:100) serum were then placed in each well, incubated for 3 h at room temperature and washed several times. Eighty $\mu$l per well of biotinylated anti-PAP IgGs at 3 $\mu$g ml$^{-1}$ in PBS containing 10% rabbit serum were then added as a second antibody for 30 min. After washing, 80 $\mu$l of avidin–peroxidase 1:5000 in PBS was added to each well for 15 min and, after washing, 80 $\mu$l of OPD were substituted and incubated 15 min in the dark. Finally, the reaction was stopped with 80 $\mu$l of sulphuric acid, 2 N, and samples were read at 492 nm. Quantification was made by comparison with a calibration curve obtained with serial dilutions of recombinant PAP.

*Statistical analysis* Data were expressed as mean±s.d. Distribution was described by the 'box and whiskers' representation, the boxes enclosing 50% of the data around the median and the whiskers extending to the points being within 1.5 times the interquartile range. The Kruskal–Wallis test was used to compare PAP concentrations between study groups. Pearson's *r* correlation was used to evaluate the correlation between individual values of PAP and values of the different tests of hepatic function.

### Results

PAP I *gene transcription in the hepatoma* HEPG2 *cell line*

*The* PAP I *gene is efficiently transcribed in hepatoma HepG2 but not in fibroblast Rat2 cells* A prerequisite to these experiments was that endogenous PAP I mRNA is expressed in HepG2 cells. This was demonstrated using a reverse transcriptase–polymerase chain reaction (RT–PCR) approach with specific human PAP I primers, as already reported (Dusetti *et al.*, 1994*b*) (not shown). To identify the DNA domains involved in the tissue-specific regulation of the *PAP I* gene expression, we dissected the 5′ flanking region of this gene by progressively deleting the upstream sequence in the 5′ to 3′ direction. The ability of these segments to drive the expression of the bacterial chloramphenicol acetyltransferase (*CAT*) gene was tested in short-term expression experiments. Plasmids carrying the progressively deleted PAP I regulatory regions (Figure 1) fused to the *CAT* gene were transfected, in parallel, into both the hepatoma HepG2 cell line and the fibroblast Rat2 cell line. The relative level of expression of these different plasmids was determined by enzymatic *CAT* assay. Progressive deletions in the 5′ to 3′ direction resulted in a stepwise decrease of *CAT* gene expression in the HepG2 cell line (Figure 1). Deletions down to the −685 position caused a 25% decrease of expression. Deletion of the sequences from −685 to −444 did not significantly affect the expression, however deletion of the sequences from −444 to −180 caused a 4–5 fold decrease of expression. Deletion of the sequences to position −118 did not have any additional effect, but a further deletion of 53 bp (to position −65) resulted in a significant reduction (about 10-fold) of the activity. In Rat2 cells, a cell line that does not express PAP I mRNA (Dusetti *et al.*, 1995*c*), deletions from −1253 to −180 did not affect *CAT* expression. However, deletion from −180 to −118 significantly increased the expression (about 5-fold). Deletions down to −118 decreased the *CAT* activity by about 5-fold. Similar results were obtained with other cell lines that do not express PAP I (data not shown). These data suggest the presence of a negative *cis*-acting sequence element between −180 and −118 in the *PAP I* gene that confers tissue-specific expression. This negative element was inactive in the hepatoma cells.

*Cell-specific activity of the −180/−118 region on heterologous promoter* Many *cis*-acting elements can regulate expression of heteropromoters (Rosenthal, 1987). To check the cell specific activity of the −180/−118 region of the PAP I promoter, this fragment was inserted upstream from the thymidine kinase promoter (Figure 2, plasmid p−180/−118PAPI/TK-CAT) driving the reporter *CAT* gene. It is important to note that this region of the PAP promoter is strongly conserved between rat and human genes as previously reported by Dusetti *et al.* (1994*b*) and others (Lasserre *et al.*, 1994). These constructs were transfected in Rat2 and HepG2 cells, and *CAT* activity was measured. The results are illustrated in Figure 2. The PAP I promoter fragment worked as a powerful negative *cis*-acting element in Rat2 cells when it was inserted upstream of the thymidine kinase promoter, in both orientations. However, this fragment was, as expected, inactive in HepG2 cells. These results confirm that the negative activity of the −180/−118 region is promoter and orientation independent, but cell specific.

*Absence of the PAP I silencer activity in the hepatoma cells* The PAP I mRNA is strongly expressed in hepatoma cells, but not in normal hepatocytes. We speculated that the silencer element described above, localised within the −180/−118 region of the PAP I promoter, was derepressed in hepatoma cells. This hypothesis was investigated by analysing the specificity of transcription of the PAP I promoter *in vitro* using nuclear extracts obtained from Rat2 cells, rat liver (in which the promoter is inactive) and hepatoma HepG2 cells (in which the promoter is active). All constructs used as *in vitro* templates contained the G-less reporter cassette (Sawadogo and Roeder, 1985). Using the vector p($C_2$AT)19, we constructed two plasmids containing regions −180/+10 and −118/+10 upstream of the G-free cassette. These templates are referred to as p−180/+10PAPI($C_2$AT)19 and p−118/+10PAPI($C_2$AT)19 respectively. As a positive control for the transcriptional competence of our extracts, we used the same G-free cassette under the direction of the Adenovirus-2 major late (AdML) promoter (referred to as pML($C_2$AT)19), which is a very strong promoter in most *in vitro* systems. The vector (p$C_2$AT)19 was used as negative control. Results are described in Figure 3. pML($C_2$AT)19 was

**Figure 1** Deletion analysis of the PAP I promoter. Relative *CAT* activity ± s.e.m. in extracts from HepG2 and Rat2 cells transfected with the corresponding plasmids. CAT activity was normalised for transfection efficiency, using the ratio of *CAT* activity to β-galactosidase activity and was expressed as a percentage of the pTK-CAT activity. Results are mean values for at least six experiments for each cell type.

**Figure 2** Identification of a silencer element in the PAP I promoter which is active in front of a heterologous promoter in Rat2 but not in HepG2 cells. Relative *CAT* activities ± s.e.m. in extracts from HepG2 and Rat2 cells transfected with the corresponding plasmids. *CAT* activity was normalised for transfection efficiency as described in Figure 1. The amount of *CAT* activity detected when pTK-CAT was transfected was arbitrarily set at 100 for each cell line. Results are mean values for at least six experiments for each cell type.

**Figure 3** *In vitro* transcription of p−118/+10PAPI($C_2$AT)19 and p−180/+10PAPI($C_2$AT)19 in HepG2 cells, rat liver and Rat2 cells nuclear extracts. Equal amounts of each plasmid (400 ng) were incubated with 30 μg of nuclear extracts from HepG2 cells, rat liver or Rat2 cells as described in Materials and methods selection. The *in vitro* transcription products were analysed on a 6% polyacrylamide−7 M urea sequencing gel. The *in vitro* transcription products from pML($C_2$AT)19 and p($C_2$AT)19 were used as positive and negative controls respectively. Specific transcription products are indicated by arrows. The amount of radioactivity incorporated into each transcript was quantitated by densitometry of the autoradiograms.

efficiently transcribed in Rat2 cells, rat liver and HepG2 cell nuclear extracts. p−118/+10PAPI($C_2$AT)19 was also transcribed with the three nuclear extracts. However, specific transcription from the p−180/+10PAPI($C_2$AT)19 was strongly repressed (about 11- and 4-fold) with nuclear extracts from Rat2 cells and rat liver respectively. In contrast, transcription of p−180/+10PAPI($C_2$AT)19 remained unaffected with extracts from hepatoma cells. These

**Figure 4** DNAase I Footprinting assays of the proximal PAP I promoter region. The DNA fragment (−378 to +10) was labelled with [$\gamma$-$^{32}$P]ATP and T4 polynucleotide kinase. Increasing amounts of nuclear extracts from HepG2 cells, rat liver and Rat2 cells were used (12, 24 or 36 $\mu$g reaction, represented by the top triangle). Control reactions (0) were incubated with 24 $\mu$g of BSA. Binding reactions and DNAase I treatment were carried out as described in Materials and methods section. G+A represents the sequences ladder. The relative positions of the DNAase I protected regions are indicated by boxes on the right of each autoradiogram.

**Table I** Correlations between serum values of PAP and some parameters of liver function in patients with hepatocellular carcinomas

| | r | P |
|---|---|---|
| Albumin | −0.54 | <0.0001 |
| Total bilirubin | 0.14 | NS |
| $\gamma$-Globulin | 0.28 | <0.03 |
| Platelets | −0.25 | <0.004 |
| Prothrombin | −0.24 | <0.01 |
| AFP | 0.12 | NS |
| ALT | −0.05 | NS |

AFP, alphafetoprotein; ALT, alanine aminotransferase.

results suggest that rat liver and Rat2 cells contain a *trans*-acting factor, interacting with the −180/−118 promoter region and repressing the transcription of the *PAP I* gene. This factor seems to be absent from hepatoma cells.

*Footprint analysis of the proximal promoter region of* PAP I As p−180/+10PAPI-CAT contains the cell-specific elements, we chose to probe the region between nucleotides −378 and +10 for specific DNA-protein interaction with nuclear protein extracts prepared from Rat2 cells, normal liver and HepG2 cells. Figure 4 shows that several DNA segments were protected by proteins present in these nuclear extracts. The location of the protected regions, relative to the transcription start site, are indicated in Figure 4.

*Immunohistochemical localisation of PAP I in HCC*

A monospecific polyclonal antibody was used to determine by immunohistochemistry which cells produced PAP I in hepatocellular carcinoma (HCC). Three primary hepatocellular carcinomas were analysed. Non-neoplastic liver tissues from the same patients were available in all three cases. The PAP I was detected in all three carcinoma samples but in none of the control liver specimen. The strong cytoplasmic staining involved only a small number of transformed hepatocytes (Figure 5a and b). In addition, transferred cells forming bile duct-like structures could also be occasionally stained (Figure 5c), whereas other cell types, such as endothelial cells, were negative. Appropriate controls established the specificity of the reaction.

*PAP serum levels*

Figure 6 shows the distribution of PAP values in the five study groups. Values in patients with chronic active or persistent hepatitis were not different from those of controls (median values at 38 and 39, compared with 34 $\mu$g $l^{-1}$). A significant increase, compared with controls, was observed in patients with liver cirrhosis ($P<0.01$, median value at 43 $\mu$g $l^{-1}$) with 4 out of 25 patients showing values over the upper threshold of normal values (90 $\mu$g $l^{-1}$. The increase was much more significant in patients with hepatocarcinoma compared with controls and also cirrhotic patients ($P<0.0001$). In the HCC group, 64% of patients had PAP values above the normal threshold. Table I shows correlations between PAP levels and a series of parameters of liver function. There was a negative correlation between PAP and albumin of prothrombin levels, two indicators of protein synthesis in liver ($P<0.0001$ and $P<0.01$ respectively) or the number of circulating platelets ($P<0.004$). There was a direct correlation with $\gamma$-globulin levels ($P<0.03$). In contrast, there was no significant correlation between PAP I serum concentrations and the parameters of cytolysis or serum levels of AFP (Table I and Figure 7).

### Discussion

In the decade that followed the original characterisation of PAP I in rat pancreatic juice, the PAP family has extended to more than a dozen proteins whose expression occurs in many tissues and shows in these tissues different patterns of regulation. Such ubiquity, suggesting an important physiological function, aroused further interest when PAP I expression was found associated with pathological situations. Important induction was reported in pancreas during development of acute pancreatitis (Iovanna *et al*., 1991*a*, *b*), and, more recently, PAP I was described in liver during hepatocarcinogenesis (Lasserre *et al*., 1992). The molecular mechanism of PAP I induction during the acute phase of pancreatitis has been extensively analysed (Dusetti *et al*., 1994*a*, *b*). The promoter allows expression in normal pancreas, the tissue specificity being controlled by a silencer

**Figure 5** Immunolocalisation of PAP I in hepatocellular carcinoma tissues. The protein was immunodetected in thin sections of hepatocellular carcinoma from cases **a**, **b** and **c** with a polyclonal antibody to the PAP I and a peroxidase–antiperoxidase secondary antibody. Original magnifications: **a** and **b**, ×700; **c**, ×280. Normal liver was used as control in **d** (×200). PAP serum values before surgical intervention were 338, 297 and 1110 $\mu g\,l^{-1}$ for cases **a**, **b** and **c** respectively.

element turned off in pancreas by *trans*-acting cellular factors (Dusetti *et al.*, 1995*c*). Activity remains very low in normal tissue and induction is triggered by factors released during inflammation, such as cytokines, which is typical of acute-phase reactants. Members of the PAP family are apparently the only pancreatic proteins that are always induced during pancreatitis (Frigerio *et al.*, 1993; Dusetti *et al.*, 1995*a*; Iovanna *et al.*, 1991*a*; Rouquier *et al.*, 1991).

The available information on PAP I expression during liver carcinogenesis suggested a more complex situation. Other proteins, such as the AFP, were also induced. However, those proteins appeared to be expressed in consequence of the dedifferentiation of the hepatocytes because they were also found in the regenerating liver and during liver development (Tsutsumi *et al.*, 1994). By contrast, PAP I was not observed in fetal or regenerating liver (Lasserre *et al.*, 1992), suggesting a mechanism specifically related to carcinogenesis. Activation of the *PAP I* gene expression during carcinogenesis does not seem to be liver specific, because most of the studied cholangiocarcinoma and stomach cancers also expressed high levels of PAP I mRNA. However, PAP I mRNA expression is not a constant factor during carcinogenes. For example, we have found no induction in any of 12 colorectal carcinoma (unpublished results).

Because control of tissue-specific expression by a silencer is an elaborate mechanism, we made the hypothesis that it had been lost during hepatic carcinogenesis. This was tested in hepatoma cells transfected with progressive deletions of the PAP I promoter fused to the reporter gene *CAT*. The silencer, corresponding to the region between nt −118 and −180 of the promoter, prevented *CAT* expression in fibroblast (Rat2) cells, as expected, and was indeed inactive in hepatoma (HepG2) cells in which abundant *CAT* expression was observed (Figures 1 and 2). This experiment would have been optimally controlled if, in normal hepatocytes, no *CAT* expression was obtained with the

**Figure 6** Serum PAP I concentrations. Serum PAP I concentration was measured in patients suffering from chronic persistent hepatitis (CPH) ($n=40$), chronic active hepatitis (CAH) ($n=41$), liver cirrhosis (Ci) ($n=25$), hepatocarcinoma (HCC) ($n=34$) or in healthy asymptomatic subjects (Co) ($n=67$). For each group, boxes enclose 50% of the values around the median (dotted line). Vertical lines extend to data within 1.5-fold the interquartile range. Values outside that range appear as dots. Values from Ci patients were significantly different from values from Co, CAH and CPH ($P<0.01$) and HCC ($P<0.0001$). Values from HCC patients were significantly different from all groups ($P<0.0001$).

same construct. Because normal hepatic cell lines are not available, we had to control our findings by using the *in vitro* transcription system developed by Sawadogo and

**Figure 7** Correlation between AFP and PAP I serum concentrations in patients with hepatocellular carcinoma. No significant correlation between AFP and PAP I serum concentrations was observed in patients with hepatocellular carcinoma ($n = 34$).

Roeder (1985). We compared the transcription capacity of nuclear extracts from normal rat liver, Rat2 cells and HepG2 cells on constructs containing the −180/−118 region of the PAP I promoter. As shown in Figure 3, the transcription obtained with HepG2 extracts was absent when using extracts from Rat2 cells and also from normal hepatocytes. Hence, hepatoma cells had probably lost the repressor *trans*-acting factor(s) present in nuclear extracts from normal hepatocytes. A preliminary characterisation of these putative *trans*-acting factors was obtained by DNAase I footprinting assay with nuclear extracts from Rat2 cells, normal rat liver and HepG2 cells. Results presented in Figure 4 revealed that similar regions of the silencer were protected with the three extracts, but the patterns of protection were different. It was concluded that several nuclear proteins bind to that region of the PAP I promoter, suggesting that the mechanism controlling the availability of the *PAP I* gene for induction was complex. Differences in the patterns observed with normal and transformed hepatocytes probably account for the derepression of the *PAP I* gene during hepatocarcinogenesis, although more detailed analysis of the factor(s) involved is required to fully understand the mechanism. Differences in footprinting pattern are not very significant between tissues expressing or not expressing PAP I mRNA, perhaps because the *trans*-acting factor responsible for silencing is present at low concentration. Alternatively, it is possible that this nuclear factor did not bind to DNA under our experimental conditions. In all cases, the mechanisms by which expression of the *trans*-acting factors controlling tissue specificity is altered during carcinogenesis remain unknown. Inactivation of the tumour-suppressor factor (Knudson, 1985; Murphree and Benedict, 1984; Hansen and Cavenee, 1987; Ueba *et al.*, 1994; El-Deiry *et al.*, 1993) and, as for AFP, de-activation of a repressor element specific to differentiated cells were suggested as possible mechanisms (Vacher and Tilghman, 1990).

Inactivation of the silencer is not sufficient to induce PAP I expression in hepatoma, but should allow other regulatory elements of the promoter to become functional. Data from Figure 1 suggest the presence of at least three positive regulatory domains, located between nt −926 and −685, between nt −444 and −181 and between nt −118 and −65. In addition, we demonstrated that induction of the PAP I promoter was indeed possible in hepatoma cells by transfecting HepG2 cells with the PAP I promoter−*CAT* construct and obtaining a 25-fold increase in *CAT* expression upon treatment with interleukin 6 (IL-6) (not shown). Further analysis, including other PAP I promoter−*CAT* constructs and footprinting assays, will be necessary to characterise the active regions and their *trans*-acting factors.

Like AFP and the CEA (Brumm *et al.*, 1989; Ma *et al.*, 1993), PAP I was found expressed in a subset of hepatocarcinoma cells only. Immunochemical analysis of three tumour specimens (Figure 5) localised the protein to transformed hepatocytes and to some oval cells whose origin, presumably ductal, remains controversial (Dunsford *et al.*, 1989). Restricted expression of those markers to certain transformed cells is probably explained by the fact that transformation does not occur simultaneously in all cells and may even proceed at different rates in different cells. Also, their expression may correspond to a certain stage of transformation and therefore be transient if further transformation results in the loss of appropriate positive *trans*-acting factors. The observation that serum PAP I levels were elevated in a majority of patients with hepatocarcinoma (Figure 6) suggested that sustained overall PAP I expresion occurred in advanced stages of cancer development. Table I shows negative correlations with circulating levels of albumin, prothrombin or platelet number and direct correlation with levels of γ-globulin. These correlations could be explained by the fact that all the patients included in this study have developed their HCCs on cirrhotic livers. It is known that cirrhosis is accompanied by decreased albumin, prothrombin and platelet number and increased γ-globulin level. However, common mechanisms regulating the PAP I mRNA induction and induction of γ-globulin remain to be explored. There was however no correlation between PAP I and AFP serum concentrations in these patients (Figure 7), indicating that the two proteins were not concomitantly expressed.

Lasserre *et al.* (1992) have described expression of PAP I mRNA in about 25% of the HCCs, whereas in our study the serum PAP levels were increased in more than 60% of the patients. These discrepancies could be explained by the fact that the PAP ELISA system is more sensitive than Northern blot. In addition, PAP serum assays reflect global synthesis of the protein by all hepatocarcinoma cells, whereas Northern blot analysis would reflect PAP I mRNA expression in the tissue sample selected for RNA extraction and analysis. However, the number of patients included in the two studies is not large enough to completely exclude sampling heterogeneity.

PAP I expression in hepatocarcinoma raised the question of its usefulness as a marker of the disease. As a first step towards addressing this question, we assayed PAP I in serum from patients with chronic hepatitis, liver cirrhosis and hepatocarcinoma because those three clinical entities often develop in that sequence. PAP I serum concentrations were in the normal range (under 90 $\mu g\ l^{-1}$) in most patients with persistent or active chronic hepatitis. In patients with cirrhosis, its average value was significantly higher than in controls and was above the threshold of normal values in 16% of the patients. The increase was more marked in hepatocarcinoma, with two thirds of the patients showing values above 90 $\mu g\ l^{-1}$, some of them being extremely high. Results from this retrospective study suggest that serum PAP I should be further evaluated as a marker of hepatocarcinoma. It would be particularly interesting to monitor the clinical evolution of cirrhotic patients with elevated serum PAP I. In addition, because AFP and PAP I inductions occur by independent mechanisms during hepatocarcinogenesis, their use as a combination of serum markers might improve hepatocarcinoma diagnosis. Previous studies have shown that AFP was elevated in 50−70% of patients (Sato *et al.*, 1993). In our sample, that proportion was 58%. However, those patients in which both AFP and PAP I were elevated amounted to 79%.

**Acknowledgements**

We are grateful to Dr G Schutz for the ptkCAT plasmid, to M Sawadogo for the p($C_2$AT)19 and pML($C_2$AT)19 plasmids and to

P Berthezene and D Rocha for the statistical and densitometric analyses respectively. N. D. is a recipient of a 'Poste Vert' INSERM fellowship. This work was supported, in part, by the French-Argentinian Cooperation Program (INSERM-CONICET). Technical assistance of P Garrido, S Barthellemy and R Grimaud is gratefully acknowledged.

### References

BARTOLI C, GHARIB B, GIORGI G, SANSONETTI A, DAGORN JC AND BERGE LEFRANC JL. (1993). A gene homologous to the *reg* gene is expressed in the human pancreas. *FEBS Lett.*, **32,** 9236-9241.

BRADFORD MM. (1976). A rapid and sensitive method for the quantitation of microgram quantities of protein utilizing the principle of protein-dye binding. *Anal. Biochem.*, **72,** 248-254.

BRUMM C, SCHULZE C, CHARELS K, MOROHOSHI T AND KLOPPEL G. (1989). The significance of alpha-fetoprotein and other tumour markers in differential immunocytochemistry of primary liver tumours. *Histopathology*, **14,** 503-513.

CHAKRABORTY C, KATSUMATA N, MYAL Y, SCHROEDTER IC, BRAZEAU P, MURPHY LJ, SHIU RP AND FRIESEN HG. (1995). Age-related changes in peptide-23/pancreatitis associated protein and pancreatic stone protein/*reg* gene expression in the rat and regulation by growth hormone-releasing hormone. *Endocrinology*, **136,** 1846-1849.

DE GROOTE J, DESMNI VJ, GEDIGK P, KORB G, POPER H, POULSEN H, SCHENER P, SCHMID M, THALER H, UHLINGER E AND WEPLER W. (1968). A classification of chronic hepatitis. *Lancet*, **2,** 626-628.

DRICKAMER K. (1988). Two distinct classes of carbohydrate-recognition domains in animal lectins. *J. Biol. Chem.*, **263,** 9557-9560.

DRICKAMER K. (1993). $Ca^{++}$-dependent carbohydrate-recognition domains in animal lectins. *Curr. Opin. Struct. Biol.*, **3,** 393-400.

DUNSFORD HA, KARNASUTA C, HUNT JM AND SELL S. (1989). Different lineages of chemically induced hepatocellular carcinoma in rats defined by monoclonal antibodies. *Cancer Res.*, **49,** 4894-4900.

DUSETTI N, FRIGERIO JM, KEIM V, DAGORN JC AND IOVANNA JL. (1993). Structural organization of the gene encoding the rat pancreatitis-associated protein. Analysis of its evolutionary history reveals an ancient divergenes from the other carbohydrate-recognition domain-containing genes. *J. Biol. Chem.*, **268,** 14470-14475.

DUSETTI N, MALLO G, DAGORN JC AND IOVANNA JL. (1994*a*). Serum from rats with acute pancreatitis expression of the PAP mRNA in the pancreatic acinar cell line AR-42J. *Biochem. Biophys. Res. Comm.*, **204,** 238-249.

DUSETTI N, FRIGERIO JM, FOX MF, SWALLOW DM, DAGORN JC AND IOVANNA JL. (1994*b*). Molecular cloning, genomic organization, and chromosomal localization of the human pancreatitis-associated protein (PAP) gene. *Genomics*, **19,** 108-114.

DUSETTI N, FRIGERIO JM, SZPIRER C, DAGORN JC AND IOVANNA JL. (1995*a*). Cloning, expression and chromosomal localization of the rat PAP III gene. *Biochemical J.*, **307,** 9-16.

DUSETTI N, ORITZ E, MALLO G, DAGORN JC AND IOVANNA JL. (1995*b*). The pancreatitis associated protein I (PAP I), an acute phase protein induced by cytokines. Identification of two functional IL-6REs in the rat PAP I promoter region. *J. Biol. Chem.*, **270,** 22417-22421.

DUSETTI N, ORTIZ E, DAGORN JC AND IOVANNA JL. (1995*c*). Identification of a transcriptional regulatory region of the rat Pancreatitis Associated Protein I (PAP I) that confers tissue specificity. *Biochem. J.*, **311,** 643-647.

EHLEN T AND DUBEAU L. (1989). Detection of ras point mutations by polymerase chain reaction using mutation-specific inosine-containing oligonucleotide primers. *Biochem. Biophys. Res. Commun.*, **160,** 441-447.

EL-DEIRY WS, TOKINO T, VELCULESCU VE, LEVY DB, PARSONS R, TRENT JM, LIN D, MERCER WE, KINZLER KW AND VOGELSTEIN B. (1993). WAF, a potential mediator of p53 tumor suppressor. *Cell*, **75,** 817-825.

FRIGERIO JM, DUSETTI N, KEIM V, DAGORN JC AND IOVANNA JL. (1993). Identification of a second rat Pancreatitis-Associated Protein. Messenger RNA cloning, gene structure and expression during acute pancreatitis. *Biochemistry*, **32,** 9236-9241.

GIORGI D, BERNARD JP, ROUQUIER S, IOVANNA JL, SARLES H AND DAGORN JC. (1989). Secretory pancreatic stone protein mRNA: nucleotide sequence and expression in chronic calcifying pancreatitis. *J. Clin. Invest.*, **84,** 100-106.

GORSKI K, CARNEIRO M AND SCHIBLER U. (1986). Tissue-specific *in vitro* transcription from the mouse albumin promoter. *Cell*, **47,** 767-776.

GRAHAM FL AND VAN DER EB AJ. (1973). A new technique for the assay of infectivity of human adenovirus 5 DNA. *Virology*, **52,** 456-467.

HANSEN MF AND CAVENEE WK. (1987). Genetics of cancer predisposition. *Cancer Res.*, **47,** 5518-5527.

IOVANNA JL, ORELLE B, KEIM V AND DAGORN JC. (1991*a*). Messenger RNA sequence and expression of rat Pancreatitis-Associated Protein (PAP), a lectin-related protein overexpressed during acute experimental pancreatitis. *J. Biol. Chem.*, **266,** 24664-24669.

IOVANNA JL, KEIM V, MICHEL R AND DAGORN JC. (1991*b*). Pancreatic gene expression is altered during acute experimental pancreatitis in the rat. *Am. J. Physiol.*, **261,** G485-G489.

IOVANNA JL, KEIM V, BOSSHARD A, ORELLE B, FRIGERIO JM, DUSETTI N AND DAGORN JC. (1993). PAP, a pancreatic secretory protein induced during acute pancreatitis, is expressed in rat intestine. *Am. J. Physiol.*, **265,** G611-G618.

IOVANNA JL, KEIM V, NORDBACK I, MONTALTO G, CAMARENA J, LETOUBLON F, LÉVY P, BERTHEZENE P AND DAGORN JC. (1994). Serum PAP levels as prognostic indicator during the course of acute pancreatitis. *Gastroenterology*, **106,** 728-734.

ITHO T AND TERAOKA H. (1993). Cloning and tissue-specific expression of cDNAs for the human and mouse homologues of rat pancreatitis-associated protein (PAP). *Biochem. Biophys. Acta*, **1172,** 184-186.

KAMIMURA T, WEST C AND BEUTLER E. (1992). Sequence of a cDNA clone encoding a rat *Reg*-2 protein. *Gene*, **118,** 299-300.

KATSUMATA N, CHAKRABORTY C, MYAL Y, SCHROEDTER IC, MURPHY LJ, SHIU RP AND FRIESEN HG. (1995). Molecular cloning and expression of peptide 23, a growth hormone-releasing hormone-inducible pituitary protein. *Endocrinology*, **136,** 1332-1339.

KEIM V, IOVANNA JL, ROHR G, USADEL K AND DAGORN JC. (1991). Characterization of a rat pancreatic secretory protein associated with pancreatitis. *Gastroenterology*, **100,** 775-782.

KEIM V, IOVANNA JL, ORELLE B, VERDIER JM, BÜSING M, HOPT U AND DAGORN JC. (1992). A novel exocrine protein associated with pancreas transplantation in humans. *Gastroenterology*, **103,** 248-254.

KNUDSON AG. (1985). Hereditary cancer, oncogenes, and anti-oncogenes. *Cancer Res.*, **45,** 1437-1443.

LASSERRE C, CHRISTA L, SIMON M, VERNIER P AND BRÉCHOT C. (1992). A novel gene (HIP) activated in human primary liver cancer. *Cancer Res.*, **52,** 5089-5095.

LASSERRE C, SIMON MT, ISHIKAWA H, DIRIONG S, NGUYEN VC, CHRISTA L, VERNIER P AND BRÉCHOT C. (1994). Structural organization and chromosomal localization of a human gene (HIP/PAP) encoding a C-type lectin overexpressed in primary liver cancer. *Eur. J. Biochem.*, **224,** 29-38.

LUCKOW B AND SCHUTZ G. (1987). CAT constructions with multiple unique restriction sites for the functional anlaysis of eukariotic promoters and regulatory elements. *Nucleic Acids Res.*, **15,** 5490.

MA CK, ZARBO RJ, FRIERSON HF AND LEE MW. (1993). Comparative immunohistochemical study of primary and metastatic carcinomas of the liver. *Am. J. Clin. Pathol.*, **99,** 551-557.

MASCIOTRA L, LECHENE DE LA PORTE P, FRIGERIO JM, DUSETTI N, DAGORN JC AND IOVANNA JL. (1995). Immunocytochemical localization of the pancreatitis associated protein in the human small intestine. *Dig. Dis. Sci.*, **40,** 519-524.

MURPHREE A AND BENEDICT W. (1984). Retinoblastoma: clues to human oncogenesis. *Science*, **223,** 1028-1033.

ORELLE B, KEIM V, MASCIOTRA L, DAGORN JC AND IOVANNA JL. (1992). Human pancreatitis associated protein. Messenger RNA cloning and expression in pancreatic diseases. *J. Clin. Invest.*, **90,** 2284-2291.

ROSENTHAL N. (1987). Identification of regulatory elements of cloned genes with functional assays. *Methods. Enzymol.*, **152,** 704-720.

ROUQUIER S, VERDIER JM, IOVANNA JL, DAGORN JC AND GIORGI D. (1991). Rat pancreatic stone protein messenger RNA: Abundant expression in mature exocrine cells, regulation by food content and sequence identity with the endocrine *reg* transplant. *J. Biol. Chem.*, **266,** 786–791.

ROY RJ, GOSSELIN P AND GUÉRIN SL. (1991). A short protocol for micro-purification of nuclear proteins from whole animal tissue. *Biotechniques*, **11,** 770–777.

SAMBROOK J, FRITSCH EF AND MANIATIS T. (1989). *Molecular Cloning: A Laboratory Manual*, 2nd ed. Cold Spring Harbor Laboratory: New York.

SATO Y, NAKATA K, KATO Y, SHIMA M, ISHII N, KOJI T, TAKETA K, ENDO Y AND NAGATAKI S. (1993). Early recognition of hepatocellular carcinoma based on altered profiles of alfa-fetoprotein. *N. Engl. J. Med.*, **328,** 1802–1806.

SAWADOGO M AND ROEDER RG. (1985). Factors involved in specific transcription by human RNA polymerase II: analysis by a rapid quantitative *in vitro* assay. *Proc. Natl Acad. Sci. USA*, **82,** 4394–4398.

SEED B AND SHEEN JY. (1988). A simple phase-extraction assay for chloramphenicol acyltransferase activity. *Gene*, **67,** 271–278.

STENBERGER LA, HARDY PH, CUCULIS JJ AND MEYER HG. (1970). The unlabelled antibody enzyme method of immunohistochemistry. Preparation and properties of soluble antigen-antibody complex (horseradish peroxidase-antihorseradish peroxidase) and its use in identification of spirochetes. *J. Histochem. Cytochem.*, **18,** 315–333.

STEPHANOVA E, TISSIR F, DUSETTI N, IOVANNA JL, SZPIRER J AND SZPIRER C. (1996). The rat genes encoding the pancreatitis-associated proteins I, II and III (*Pap1, Pap2, Pap3*), and the lithostathine/pancreatic stone protein/regeneration protein (*reg*) colocalize at 4q33-34. *Cytogenet. Cell. Genet.*, **72,** 83–85.

TACHIBANA K, MARQUARDT H, YOKOYA S AND FRIESEN HG. (1988). Growth hormone-releasing hormone stimulates and somatostatin inhibits the release of a novel protein by cultured rat pituitary cells. *Mol. Endocrinol.*, **2,** 973–978.

TERAZONO K, YAMAMOTO H, TAKASAWA S, SHIGA K, YONEMURA Y, TOCHINO Y AND OKAMOTO H. (1988). A novel gene activated in regenerating islets. *J. Biol. Chem.*, **263,** 2111–2114.

TSUTSUMI T, IDO A, NAKAO K, HAMASAKI K, KATO Y, OHTSURU A, NAKATA K, TAMAOKI T AND NAGATAKI S. (1994). Reciprocal regulation of alpha-fetoprotein and albumin gene expression by butyrate in human hepatoma cells. *Gastroenterology*, **107,** 499–504.

UEBA T, NOSAKA T, TAKAHASHI JA, SHIBATA F, FLOKIEWICZ RZ, VOGELSTEIN B, ODA Y, KIKUCHI H AND HATANAKA M. (1994). Transcriptional regulation of basic fibroblast growth factor gene by p53 in human glioblastoma and hepatocellular carcinoma cells. *Proc. Natl Acad. Sci. USA*, **91,** 9009–9013.

VACHER J AND TILGHMAN SM. (1990). Dominant negative regulation of the mouse a-fetoprotein gene in adult liver. *Science*, **250,** 1732–1735.